# Sexual Dimorphism in Glucocorticoid Stress Response

**DOI:** 10.3390/ijms22063139

**Published:** 2021-03-19

**Authors:** Marie-Pierre Moisan

**Affiliations:** Université Bordeaux, INRAE, UMR1286, Nutrineuro, 33076 Bordeaux, France; Marie-Pierre.Moisan@inrae.fr; Tel.: +33-5-5757-9214

**Keywords:** sex dimorphism, glucocorticoid, stress, cortisol, steroids

## Abstract

Chronic stress is encountered in our everyday life and is thought to contribute to a number of diseases. Many of these stress-related disorders display a sex bias. Because glucocorticoid hormones are the main biological mediator of chronic stress, researchers have been interested in understanding the sexual dimorphism in glucocorticoid stress response to better explain the sex bias in stress-related diseases. Although not yet demonstrated for glucocorticoid regulation, sex chromosomes do influence sex-specific biology as soon as conception. Then a transient rise in testosterone start to shape the male brain during the prenatal period differently to the female brain. These organizational effects are completed just before puberty. The cerebral regions implicated in glucocorticoid regulation at rest and after stress are thereby impacted in a sex-specific manner. After puberty, the high levels of all gonadal hormones will interact with glucocorticoid hormones in specific crosstalk through their respective nuclear receptors. In addition, stress occurring early in life, in particular during the prenatal period and in adolescence will prime in the long-term glucocorticoid stress response through epigenetic mechanisms, again in a sex-specific manner. Altogether, various molecular mechanisms explain sex-specific glucocorticoid stress responses that do not exclude important gender effects in humans.

## 1. Introduction

Glucocorticoid hormones (GC), end products of the hypothalamic-pituitary-adrenal axis (HPA) are steroids produced by the adrenal glands that regulate many functions in the body including inflammation, energetic metabolism, growth, as well as mood and memory. GCs are also highly responsive to stress with both rapid and enduring effects on the body. The molecular mechanisms responsible for these effects have been largely investigated in animal models. Sexual dimorphism in baseline and stress-induced GC action are well described and could explain the sex differences observed in the prevalence of stress-related diseases. In this selective review we first briefly review the stress response system, then we present data supporting a role for sex chromosomes into sex differences followed by evidence of gonadal influences across development in shaping the sex-specific GC sensitivity. Finally we give examples of long-lasting effects of early-life stress on GC stress response according to sex. Although these various genetic, developmental, and programming effects are presented separately they obviously synergize within individuals either to exacerbate or to reduce sexual dimorphism.

## 2. Stress Response System, the Hypothalamic-Pituitary-Adrenal Axis, and GC Hormones

**Figure 1:**
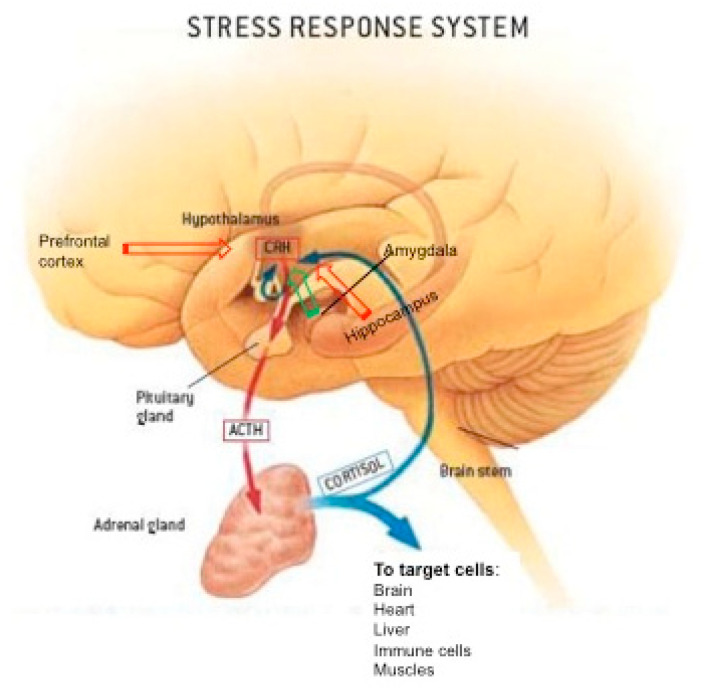
Representation of the hypothalamic-pituitary-adrenal (HPA) axis, leading to the secretion of glucocorticoid hormones (cortisol in humans, corticosterone in rodents). Prefrontal cortex and hippocampus regulating negatively the HPA axis have a red arrow whereas amygdala that regulates the HPA axis positively has a green arrow pointing to the hypothalamus. Glucocorticoids regulate their own synthesis by feedback controls (thin blue arrow).

A loss of control, a threat, an uncertain or unpredictable situation, trigger a stress response. This arousal activates the amygdala (a group of neuronal nuclei located in the temporal median lobe of the brain) and leads to the release of the neuropeptide corticotropin releasing factor (CRF) from the paraventricular nucleus of the hypothalamus. CRF together with arginine vasopressin (AVP) is transported through the hypophyseal portal capillary system from the hypothalamus to the pituitary where they stimulate the blood release of adrenocorticotropin hormone (ACTH), which in turn activates in the adrenal glands the synthesis of GC hormones (cortisol in human and corticosterone in rodents) and their secretion in the bloodstream [1,2] (Figure 1). In blood, most GCs circulate bound to corticosteroid binding globulin (CBG) with high affinity and to albumin with low affinity. Only free GCs will bind to their receptors in target cells such that a pool of CBG-bound GCs circulate in an inactive form and is released upon cleavage of the CBG-GC complex making CBG a reservoir and delivery molecule [3]. GCs have two types of receptors, the mineralocorticoid receptor (MR) presenting a high affinity for GCs but an expression restricted to some tissues and the GC receptor (GR) having a ten-time lower affinity but a much wider distribution in the body. Both GR and MR are nuclear receptors located in the cytosol when unbound and translocated to the nucleus upon binding to GCs where dimers of the hormone-receptor complex will activate the expression of downstream genes by binding to GC response elements (GRE) present in the DNA sequence or by repressing other genes by preventing other nuclear factors, such as nuclear factor kappa B (NFKB) or activation protein 1 (AP-1), to activate their target genes through protein–protein interaction [4,5]. Membrane MR and GR have also been reported permitting fast actions of GC within the brain [6,7]. GC hormones regulate their own synthesis by a negative feedback control on CRF transcription in hypothalamus or ACTH expression in pituitary. The molecular mechanisms for CRF gene inhibition by GR are still debated. Some studies reported GR binding to a negative GRE in the promoter region of the CRF gene [8] while others found that inhibition of CRH by GR involves protein–protein interaction with CREB (C-AMP Response Element-binding protein), preventing CREB binding to the CREB response element present in CRF promoter [9]. As for ACTH down-regulation in the pituitary, it was shown that GR binds to a GRE within the pro-opiomelanocortin (POMC) gene (precursor of ACTH peptide) but also GR interacts with nuclear receptor subfamily 4 group A member 1(NR4A1) transcription factor to prevent POMC induction [10]. Furthermore, limbic structures such as the hippocampus and the prefrontal cortex do contain GRs and are exerting a tonic inhibition on the hypothalamic-pituitary-adrenal axis that is relieved during stress [11]. Intra-adrenal GR-mediated negative feedback has also been described, involving stimulation of the steroidogenic co-repressor Nuclear Receptor Subfamily 0 Group B Member 1 (NR0B1) and prevention of NR4A1 binding on the promoter of Star gene [12]. Finally, GCs can be synthesized locally by the enzyme 11b-hydroxysteroid dehydrogenase type 1 which regenerate GCs from their inactive metabolite. This enzyme is present in liver, adipose tissue, and brain and can represent up to 40% of circulating GCs. Conversely, 11 β-hydroxysteroid dehydrogenase type 2 is an enzyme metabolizing GCs which is present in aldosterone target tissues like kidney to prevent GC binding to MR [13]. GCs are irreversibly metabolized in the liver thanks to the enzymes 5α and 5β reductase.

## 3. Genetic Causes of Sexual Dimorphism

As soon as conception, male and female mammals differ by the presence of sexual chromosomes in their cells, XX in females and XY in males. The two pseudo-autosomal regions (PAR1 and PAR2) located at the tips of chromosomes X and Y share the same genes which are thus inherited as autosomal genes and allow the X and Y chromosomes to pair and properly segregate during meiosis in males [14]. There are about 55 genes on Y chromosome and 29 found in the PARs, so the rest of them (~25 genes) are expressed only in males. One of the X chromosome of females is inactivated by epigenetic mechanisms to avoid double dose of X-linked genes compared to males [15]. This is true for most genes but some genes escape to the X inactivation specifically during development. Furthermore, the inactivation of maternal versus paternal X chromosome is random in females, which also impact gene expression when genes are under genomic imprinting specific to the maternal or paternal allele [16]. The X chromosome in male is necessarily from maternal origin, so more heterogeneity of expression could be observed for some X chromosome-linked traits in females. Interestingly, X-linked genes are more highly expressed in the brain versus other tissues [17] so those that escape X-inactivation are good candidates for sexual differences in brain development that may impact the HPA axis regulation. A possible example is given by the *Frm1* gene for which inactivation is responsible of fragile X syndrome. Affected males have more severe symptoms of mental retardation but also of social anxiety and they display higher cortisol levels [18]. A direct effect of *Frm1* gene on cortisol levels sex differences is not yet elucidated but may rise from the involvement of *Frm1* in dendritic spines formation and maintenance in brain regions controlling the HPA axis regulation.

Another way sex dimorphism may appear from, is when parents differentially influence gene expression in the brain of daughters versus sons. A genome-wide study identified preferential selection of the maternally inherited X chromosome in glutamatergic neurons of the female prefrontal cortex. Such sex-specific genomic imprinting inheritance is not restricted to X-linked genes as 347 autosomal genes with sex-specific imprinting features were also identified in adult brain of offspring, including prefrontal cortex, hippocampus and hypothalamus [19]. These data are particularly interesting given that these cerebral regions strongly regulate the HPA axis and the release of GCs under stress.

## 4. Sex Dimorphism Related to Gonadal Hormones across Development

### 4.1. Neonatal Period

Around birth the expression of the Y-linked *Sry* gene triggers testes differentiation with the production of testosterone in male rodents as a prenatal surge in late gestation and a second surge that occurs immediately after birth [20]. In humans this testosterone surge is present postnatally only [20,21]. Circulating neonatal testosterone has profound effects on organ development such as liver and brain that in turn will sculpt HPA activity and reactivity after puberty and result in sex dimorphism.

The first 2 postnatal weeks in rodents have been termed the “stress hypo-responsive period” because of very low basal and stress-induced corticosterone levels. In human this period may correspond to childhood [22]. This low activity of the HPA axis is due in large part to the limited expression of *SerpinA6* gene in liver, leading to the production and secretion of CBG in plasma. Indeed, CBG levels set the level of basal circulating GCs in great part because it increases GCs half-life in plasma by preventing their plasma clearance as observed in CBG knock-out mice [23]. Also, by sequestering GCs in inactive forms, CBG stimulates adrenal gland growth and function in order to maintain free CORT levels [24]. This low activity of the HPA axis after birth is also reflected in the low expression of 11β-hydroxysteroid dehydrogenase type 1 (*Hsd11b1*), that regenerate GCs from their inactive metabolite and 5α-reductase type I (*Srd5a1*) which irreversibly inactivate GCs before clearance [24].

Neonatal testosterone surge will influence HPA axis sex dimorphism by indirectly modulating these 3 hepatic genes *SerpinA6*, *Hsd11b1*, and *Srd5a1*, through the growth hormone-insulin growth factor I (GH-IGF-1) pathway that regulate these genes. Indeed, in rodents, testosterone exposure during the first days of life determines the sex-specific pattern of GH secretion in adulthood which show more frequent and higher amplitude secretory pulses in males compared to females [25]. This GH secretory pattern is regulated in hypothalamic neurons by somatostatin and growth hormone-releasing hormone that inhibits and stimulates GH release from the anterior pituitary respectively. Testosterone crosses the blood-brain barrier to reach the brain where it is converted to estradiol by the enzyme aromatase. Prevention of testosterone surge by gonadectomy at birth feminizes GH secretory pattern in males while neonatal exposure to testosterone or exposure to exogenous estradiol in females masculinizes GH release. Interestingly, continuous infusion of GH (female pattern) increases plasma CBG in hypophysectomized males and females while intermittent GH exposure (male pattern) has no effect or suppresses CBG levels in females [26]. Thus, sex differences in CBG levels that emerge around puberty are due in large part to sex-specific GH secretory pattern that is determined by neonatal testosterone. Similarly, GH regulates differentially *Hsd11b1* and *Srd5a1* in male and female liver, accentuating sexual dimorphism in GC levels appearing after puberty (reviewed in [24]).

In both humans and rodents, several brain regions involved in stress and CRF-mediated HPA axis regulation, such as the prefrontal cortex, amygdala, hippocampus, and hypothalamus undergo significant maturation in the perinatal period and during childhood and adolescence. These areas also demonstrate significant sex differences in both structure and function into adulthood. Besides sex chromosomes, gonadal hormones do affect the rates of maturation of these brain regions between males and females, and thereby ultimately determining the GC stress sensitivity during adulthood [27].

Neonatal testosterone, converted to estradiol, drives masculinization, an active process of neurodevelopment including neurogenesis, cellular differentiation, axon guidance, synaptic pruning, apoptosis, and phagocytosis [21]. Masculinization has been associated with male-specific reproductive behavior but it also impacts HPA functioning. Indeed, gonadectomy around birth in males increases basal and stress-induced corticosterone levels in adults [28] while a single injection of testosterone in females on the day of birth decreases the amplitude and frequency of corticosterone pulsatile secretion as well as stress-induced levels [29]. Additionally, the inhibition of aromatase enzyme in male neonates increases stress-induced corticosterone once rat are adults and also increased c-fos mRNA, a marker of neuronal activation, in the paraventricular nucleus of the hypothalamus and in hippocampus [30]. Thus, neonatal testosterone, through its conversion to estrogen and via estrogen receptor alpha (ERα) signaling has a suppressive effect on GC stress response in adult rodents by organizational effect in liver and in brain.

Humans, like rodents are exposed to a gestational testosterone surge and testosterone aromatization does occur but masculinization of the brain seems to be mediated by androgen receptor (AR) signaling. Indeed, men with defective aromatase enzyme conserved male-specific behavior while men with AR mutations, leading to complete androgen insensitivity syndrome, exhibit female-typical behavior [31]. A direct effect of neonatal testosterone on GH release has not been reported in men where GH secretion pattern are less pronounced compared to the rat. Nevertheless, higher nocturnal pulses of GH in men were reported whereas women had more continuous GH secretion and more frequent GH pulses that are of more uniform size [32]. Further, GH-deficient adults with GH replacement display a negative correlation between plasma IGF1 and CBG (decreased by 30%) suggesting down-regulation of CBG by IGF-1, but no significant effect on total cortisol was detected [33].

### 4.2. Puberty Period

A second critical period for the organizational influence of gonadal hormones on the HPA axis is puberty. Puberty is marked by many changes in neuroendocrine processes, one of which is the shift in stress reactivity exhibited by the HPA axis. Prepubertal rats of both sexes show an extended hormonal stress response compared to adults (~40 min longer) and do not habituate to repetitive stress [34]. Studies in male rats showed that prepubertal gonadectomy results in an HPA axis that is less responsive to androgen treatment in adulthood, i.e., lower inhibition by androgens of corticosterone secretion and of frequency and amplitude of corticosteroid pulses. In contrast, animals that were gonadectomized in adulthood showed a normal response to testosterone replacement [35]. Furthermore, stress-induced increase in CRF and AVP mRNAs in the hypothalamus are suppressed by testosterone only in the animals that were castrated in adulthood. Thus, testosterone exposure during the prepubertal period is important for the masculinization of GC responses to stress in adulthood. Unlike testosterone, pubertal estradiol exposure is not essential for sculpting female-typical HPA responses in adulthood since ovariectomy before or after puberty will result in the same stimulatory effect of estradiol on basal and stress-induced corticosterone [36].

### 4.3. Adulthood

After these two organizational periods, adulthood is characterized by activation of sex differences owing to elevated concentrations of gonadal hormones. After puberty female rodents display higher levels of circulating GCs at rest and after stress. Of note, free active GCs are equivalent to males because of higher CBG levels [23]. In humans, inconsistent data are reported in the literature about sex differences in cortisol levels, probably because of the difficulty to measure cortisol in controlled conditions. For example, females were found to have higher free cortisol levels than males, as in rodents, in one study with 1258 participants [37] but the opposite was found in another one where there was no differences at rest and a higher response after a social stress in men [38]. A meta-analysis for post-stress cortisol levels concluded that men had higher cortisol levels than women but the sex differences could be explained by variations in methodologies [39]. Increased basal levels of total GCs in females are largely mediated by CBG since in Cbg ko mice, GCs are very low in both sexes and 24 h urine corticosterone levels become equivalent in males and females [23]. Similarly, in human totally deficient in CBG, cortisol concentrations are very low and do not differ between sexes [40]. Sex differences are found also centrally with female rodents presenting higher levels of CRF and AVP mRNA in the hypothalamus as well as increased POMC expression in pituitary compared to males [41]. Feedback regulation of the HPA axis is less efficient in females and is associated with lower binding of GR in the hypothalamus as well as in the pituitary [42]. Furthermore, the analysis of hypothalamus-specific ko of GR in mice showed that negative feedback is disrupted in males but not in females suggesting that the site of feedback control is elsewhere in females [43].

### 4.4. Molecular Aspects

Androgens down-regulate both basal and stress-induced GC levels through central and peripheral actions while estrogens have a stimulating effect not only by increasing CBG but also by impairing GC feedback or centrally inducing the HPA axis. This effect of estrogens depends on the presence or absence of progesterone that indirectly counteracts estrogen stimulating action [27,44].

Testosterone acts through AR or through ERα or ERβ after testosterone conversion to estradiol or successive conversion of testosterone to dihydrotestosterone and 5α androstane 3β,17β diol (3β-diol). Estrogens bind to both ERα and ERβ. As GR and MR, AR, Erα, and ERβ are members of the family of steroid nuclear receptors that are ligand-inducible transcription factors; they have a centrally located DNA-binding domain (DBD) which is connected via a hinge region to a carboxyterminal ligand-binding domain (LBD) and an amino-terminal activation function (NTD). The DBD consists of two zinc-coordinating modules and forms the signature domain of the nuclear-receptor family. Nuclear receptors remain in an inactive form in the cytoplasm when unbound and then shuttle to the nucleus upon hormone binding to activate downstream target genes on specific hormone response element in the DNA sequence or to down-regulate genes by protein–protein interaction with other transcription factors. Membrane ER receptors have also been described allowing non-genomic rapid actions of estrogens through second messenger pathways and ion channels [45].

Testosterone influence on HPA axis tone is blocked by intra-cerebro-ventricular infusion of a 5α-reductase inhibitor, which converts testosterone to the more potent and non-aromatizable androgen, dihydrotestosterone (DHT), suggesting central effects of the hormone. Anatomically, the action of testosterone on HPA drive is thought to occur via the bed nucleus of the stria terminalis (BNST) in addition to the paraventricular nucleus (PVN) of the hypothalamus since AR are particularly abundant in BNST neurons that projects to the PVN. Androgen response elements are present in the CRF gene [46]. HPA suppression by androgens is obtained by a decrease of CRF gene and an increase of its receptor CRFR1 in hypothalamus [47]. This central effect explains increased POMC mRNA as well as hypertrophic adrenal glands observed in AR knockout mice but decreased GR in the pituitary was also detected and appeared to be regulated by androgen although no classical androgen response element (ARE) was found in the GR gene [47]. Androgens can also interact with ERβ via conversion of testosterone to estradiol or through 3β-diol, which has a high affinity for ERβ, but very low affinity for AR. Erβ are abundant in the PVN and specific activation of these receptors suppresses stress-induced ACTH and GC secretion as well as c-Fos expression in the PVN [48]. Mechanistically, Erβ-3β-diol complex was shown to bind to an estrogen response element (ERE) within the promoter of the AVP gene, thereby inhibiting ACTH release from pituitary [49]. Additionally, Erβ-3β-diol complex also binds to an ERE in the promoter of the oxytocin gene [50] including in humans [51] to induce oxytocin production which is known to attenuate stress-induced ACTH and GC secretion.

ERα are not present in the PVN so direct influence of gonadal hormone on CRF neurons of the PVN are excluded. However, ERα are abundant in the peri-PVN where they mediate the inhibiting influence of estradiol on the negative feedback of GC thereby up-regulating HPA drive. Indeed, estradiol injection directly within the hypothalamus prevents dexamethasone (a GR agonist) suppression of corticosterone release in rats via ERα but not ERβ [1]. Additionally, the CRF gene contains ERE and ERα as well as ERβ were shown to induced CRF in neuroblastoma cell lines [52]. However, ERα and ERβ were found to regulate CRF-BP gene expression in opposite ways, ERα inducing and ERβ repressing CRF-BP [53]. Although CRF-BP is often reported to inhibit CRF by preventing its binding to CRFR1 in the pituitary, transgenic mice with overexpression of CRF-BP display elevated CRF levels and no change in ACTH and GC levels [54]. Thus the ERα and ERβ regulation of CRF-BP fits with a positive effect of ERα and a suppressive one of ERβ on CRH. The influence of testosterone or estradiol on adrenal steroidogenesis was studied by transcriptomic analyses comparing rat controls to rats gonadectomized or gonadectomized and replaced by either testosterone (males) or estradiol (females). Although the effect of these hormonal manipulations resulted in the expected outcome for corticosterone levels, unexpected upregulation of many genes was found after testosterone replacement, including genes associated with lipids and cholesterol metabolism while estradiol replacement inhibited the expression of numerous genes, mainly those involved in tumor necrosis factor alpha (TNFα) signaling through NFKB [55]. Elevated CBG levels in females are ensured by estrogen stimulation of *SERPINA6* gene through ERα binding in its promoter region [56,57,58], which explains very high cortisol and CBG levels in women taking estradiol contraceptive pills or during pregnancy.

Finally, progesterone acts on the HPA through its metabolite 3α-5α tetrahydroprogesterone more commonly known as allopregnanolone. Allopregnanolone is a positive allosteric modulator of ϒ-aminobutyric acid receptors (GABA_A_) through which it inhibits CRF neurons within the PVN, consequently decreasing CRF and GC release, in particular after an acute stress [59]. Allopregnanolone is induced by stress in both males and females through adrenal steroidogenesis but the additional progesterone levels released from ovaries are responsible for a part of the sex dimorphism in GC stress response.

## 5. Sex-Specific Programming Effects of Stress on the HPA Axis

Elevations in GCs that result from environmental stressors or exogenous GCs can have programming effects on brain structure regulating the HPA axis when the exposure occurs during sensitive periods. Two sensitive periods have been identified, perinatal time and adolescence where intense brain development occurs.

### 5.1. Perinatal Environment

Maternal stress has been shown to alter the development and sensitivity of offspring HPA stress axis in the long-term and in a sex-specific manner, with increased male vulnerability in particular when the stress happens early in pregnancy. Fetal exposure to maternal GCs is known to be regulated by the placental GC barrier ensured by various mechanisms including 11β-hydroxysteroid dehydrogenase type 2 (11β-HSD2) which metabolize GCs to inactive forms (cortisol to cortisone in human, corticosterone to 11-dehydrocorticosterone in rodents), and GR isoforms such as GRβ conferring GC resistance. The placenta expresses the fetal genetic sex [60] which explains sex-specific effect of maternal stress. Considering that the newborns are brought up by mothers, that are themselves stressed, it is more appropriate to define the stress as perinatal rather than simply prenatal, and to consider postnatal maternal factors as well.

Animal models of maternal stress across a diverse range of species, mainly mice [61], rats [62], and guinea pigs [63], have demonstrated that prenatal stress increases offspring GC stress sensitivity. A recent phylogenetic meta-analysis across 14 different vertebrate species reported positive correlation between prenatal stress and dysregulated GC sensitivity, in particular in the GC feedback mechanism [64], underlying the evolutionary conserved mechanisms involved and therefore the usefulness of animal models for studying prenatal programming of the HPA axis in humans.

Whenever both sexes were analyzed in the same study, sex differences emerged. For example, in the early prenatal stress model developed by Bale’s group, in which pregnant female mice are exposed to chronic stress during the first week of gestation, male offspring were found to have a heightened GC response to stress while females were not affected. By transcriptomic analysis of placenta at various gestational stages, the gene encoding the enzyme O-linked N-acetylglucosamine transferase (OGT) was identified as a biomarker of prenatal stress as its mRNA expression was decreased by prenatal stress and it was differentially regulated in male and female offspring [65]. In fact, OGT is a X-linked gene escaping X-inactivation so down-regulation by prenatal stress has more incidences in males since females hold two copies of the gene. By deleting OGT specifically in placenta, the causal role of OGT in male offspring’s increased stress sensitivity was demonstrated [66]. Recently, the same group showed that one mechanism whereby OGT confer stress resilience in females is by up-regulating the repressive epigenetic mark H3K27me3 in placenta as demonstrated by the down-regulation of H3K27me3 in females through genetic manipulation which render them vulnerable to prenatal stress with increased GC response to stress as found in males [67].

In a nice translational study [68], blood mRNA expression of the gene GC-induced leucine zipper (GILTZ encoded by *Tsc22d3* gene) was found decreased in subjects with post-traumatic stress disorder (PTSD), and especially in those that presented traumatic events in childhood, both in males and females. However, only in males GILTZ mRNA were significantly negatively correlated with the lifetime total number of traumatic events experienced. Also, in males only, GILTZ mRNA were significantly negatively correlated with the percentage of methylation of an intronic CpG island within the GILTZ gene DNA sequence, suggesting regulation by methylation. To better understand these effects, mouse models were used to further understand the mechanism by which multiple lifetime traumas affect GILZ expression and its epigenetic regulation or by which one early-life trauma predisposes to additional trauma later in life. The “one early-life trauma” consisted of a prenatal stress induced by brain overexpression of CRF during late gestation and the model of multiple trauma was the same prenatal stress plus a protocol of “stress enhanced fear learning” at adulthood which induces PTSD-like symptoms in a subset of mice. As in humans, prenatal stress predisposed male but not female mice to PTSD symptoms with reduced GILTZ mRNA levels in amygdala correlated with increased methylation in a GRE of the GILTZ gene DNA sequence. GILTZ gene being X-linked, females are less vulnerable to changes in GILZ levels after trauma.

Another example of prenatal stress in human is given by the study of pregnancy complicated with asthma [69] which results in increased levels of maternal cortisol. Placental 11β-HSD2 activity was found reduced in male and female placentae leading to increase exposure to cortisol in both sexes but females only showed reduced birth weight, which is believed to be a consequence of elevated cortisol exposure. It appears that male placenta from pregnancy complicated by asthma display a state of GC resistance owing to the expression of GRβ and to a low transactivational isoform GRαD1 [70]. CRF gene expression was also up-regulated in female but not in male placenta. The authors proposed that female adapt to the presence of maternal asthma through suppression of their immune pathways via increased cortisol exposure at the expense of fetal growth but increased survival, whereas male favor growth via a reduced sensitivity to cortisol although it leads to increased risks of preterm birth or stillbirth.

### 5.2. Adolescent Period

Sex-specific long-term influences of adolescent stress exposure on adult responsiveness are highly dependent on the type of stressor experienced, the stage of adolescent development during which the experience occurred, and species [34]. In rats, chronic stress during adolescence was found to affect females with sustained behavioral effects lasting until adulthood and impaired corticosterone response to acute stress while males showed no changes either in adolescence or at adulthood [71]. Further investigations of the same group revealed sex differences in baseline expression of GR co-chaperones and modulators that were exacerbated by the combination of chronic stress and acute stress challenge. More precisely, females presented lower peptidyl-prolyl isomerase D (Ppid) mRNA in hippocampus at baseline but Ppid- and BCL-2-associated athanogene (Bag-1) is induced and GR translocation to the nucleus increased by chronic stress in females only. In response to an acute stressor GR, BAG-1, and FK506 binding protein 51 (FKBP5) are up-regulated 30 min after in females whereas males show changes in GR, FKBP5, and nuclear receptor coactivator 1 (SRC-1) observed only 120 min after the challenge. These data suggest enhanced GR sensitivity in chronically stressed adolescent females soon after acute stress that appeared at later time points in males. Interestingly, Ppid mRNA levels correlated with the increase in estradiol concentrations in chronically stressed but not control females, underlying the interaction between sex steroid and chronic stress in shaping GC signaling [72]. Independent studies confirmed increased vulnerability of females for chronic stress during adolescence leading to blunted or increased HPA axis reactivity depending on the type and intensity of the chronic stress [73,74,75,76,77].

Human studies are lacking to assess sex-specific programming effect of stress during adolescence, although stressors specific to this period of life are important [78]. An important difference with rats is that human hippocampus is fully developed by 2 years of age whereas rat hippocampus continues to develop until adulthood rendering this region more vulnerable to stress in adolescent rat. In both species, the frontal cortex develops well into adolescence and amygdala even further until late twenties in humans. Thus, prefrontal cortex is thought to be particularly sensitive to adversity in adolescents [22]. The effects of acute stress exposure on prefrontal cortex and its connectivity with amygdala and ventral striatum, regions also under active development during adolescence, are reviewed in [78]. The data provide support that the adolescence is vulnerable to changes in connectivity between brain regions important for stress reactivity with some sex-specific effects.

**Figure 2:**
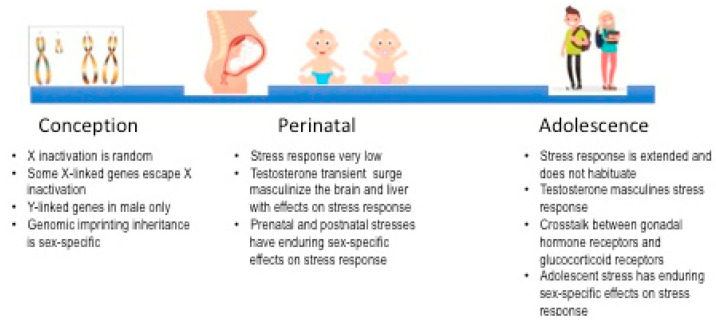
Genetic, organizational, and programming effects involved in sexual dimorphism of glucocorticoid stress response across development.

The main findings explaining glucocorticoid stress differences in adults are summarized in Figure 2.

## 6. Discussion

Sex differences in GC stress responses have been largely studied because dysregulation of GCs (hypo or hyperactivity) is a hallmark of many stress-related diseases, including psychiatric disorders, inflammatory and autoimmune diseases, or metabolic troubles [79,80]. While we have accumulated an impressive body of knowledge in the last 50 years about stress and GC biology, we have barely scratched the surface concerning sex differences. Until recently, most preclinical studies were done in males only and clinical studies were not often designed to properly compare male and female subjects. This is progressively changing now that inclusion of both sexes in research projects is promoted, if not compulsory, by the funding agencies of many countries [81].

In this review that does not intend to be exhaustive, I have summarized key ideas and findings underpinning sexual dimorphism in GC stress responses at different stages of development. Of note, the body adaptations to the environment are cumulative across the life span, and its functioning at any later point in life is the result of experiences and epigenetic alterations that take place during gestation, and throughout postnatal development. Not reviewed here are recent studies highlighting the influence of stress before conception, for example extracellular vesicles that transmit information about stress experience to sperm which could lead to sex-specific programming effects in offspring [82].

Although significant advances have been made, there are still a number of open questions remaining. One of them concerned the consequences of differential GC responses in terms of free GCs. Indeed, as pointed out in this review only free GCs are active on the body but this is rarely measured in rodents and in humans it depends whether cortisol is measured in saliva, where only free GCs are present contrary to plasma. The dynamics of free GC were specifically measured in rats with microdialysis probes by comparing free GCs in hippocampus after either a moderate stress (novel environment) or after a high-intensity stress (forced swim). As expected the forced swim stress led to higher free corticosterone levels in hippocampus compared to the moderate stress but no sex differences were observed either in the peak response or in the AUC of the free corticosterone response to both novelty and forced swim. The only change was a slightly more rapid corticosterone elevation in females for the forced swim [83]. Using isotopic dilution we also measured free GCs after forced swim in male and female mice and confirmed no sex differences in free GC levels [23]. These data underlie the importance of measuring free in addition to total GCs before interpreting sex differences.

Gender effects are also often overlooked in human research although they contribute largely to the sexual dimorphism in GC stress response. Gender roles are socially constructed characteristics of men and women contrary to pure sex differences that are only biological. In animal, gender roles do not exist but in human sex and gender effects are combined as soon as an individual is socialized. Gender effects in stress response have been first revealed in a study comparing men and women cortisol response after different types of social stress. In the first one, the subjects are submitted to a fictitious job interview with an unpleasant jury. Males showed on average a higher cortisol response than females to this test while in another test based on social rejection from a group, opposite responses were observed, with females displaying higher cortisol stress response. The authors concluded that gender role may explain these results as males are generally more affected by stress related to achievement while females would be more sensitive to stress related to social rejection [84]. Some authors are going further, evoking the critical role of gender socialization during childhood in shaping brain development and GC stress response by encouraging boys to be more active, self-centered, and autonomous while girls are more educated to provide care to others and are less valorized for autonomy. These social interactions would be translated by epigenetic mechanisms in differential hormonal and neuronal functioning in the developing brain [85]. While further studies are needed to confirm this hypothesis, human stress studies would highly benefit from competences of sociologists to evaluate the role of gender in explaining observed sex differences as pointed out in an editorial of *Nature* journal [86] *Life stresses!.*
